# Supplementary material for: DTVF: A User-Friendly Tool for Virulence Factor Prediction Based on ProtT5 and Deep Transfer Learning Models
Source: Genes (Basel). 2024 Sep 5;15(9):1170. doi: 10.3390/genes15091170 (PMC11430887; doi:10.3390/genes15091170)
Supplement: Supplementary file 1 [file genes-15-01170-s001.zip › Figure S1 - demo_embedding.pdf]

protT5
output
protT5_checkpoint
sec_struct_checkpoint
sample_data

test.fasta 2024/8/4 20:26

没有预览。

1 个项目 选中 1 个项目 340 KB

[3] 6 秒

```
Requirement already satisfied: torch in /usr/local/lib/python3.10/dist-packages (2.3.1+cu121)
Requirement already satisfied: transformers in /usr/local/lib/python3.10/dist-packages (4.42.4)
Requirement already satisfied: sentencepiece in /usr/local/lib/python3.10/dist-packages (0.1.99)
Requirement already satisfied: h5py in /usr/local/lib/python3.10/dist-packages (3.11.0)
Requirement already satisfied: filelock in /usr/local/lib/python3.10/dist-packages (from torch) (3.15.4)
Requirement already satisfied: typing-extensions>=4.8.0 in /usr/local/lib/python3.10/dist-packages (from torch) (4
Requirement already satisfied: sympy in /usr/local/lib/python3.10/dist-packages (from torch) (1.13.1)
Requirement already satisfied: networkx in /usr/local/lib/python3.10/dist-packages (from torch) (3.3)
Requirement already satisfied: jinja2 in /usr/local/lib/python3.10/dist-packages (from torch) (3.1.4)
Requirement already satisfied: fsspec in /usr/local/lib/python3.10/dist-packages (from torch) (2024.6.1)
Requirement already satisfied: nvidia-cuda-nvrtc-cu12==12.1.105 in /usr/local/lib/python3.10/dist-packages (from t
Requirement already satisfied: nvidia-cuda-runtime-cu12==12.1.105 in /usr/local/lib/python3.10/dist-packages (from
```
