# Supplementary material for: DTVF: A User-Friendly Tool for Virulence Factor Prediction Based on ProtT5 and Deep Transfer Learning Models
Source: Genes (Basel). 2024 Sep 5;15(9):1170. doi: 10.3390/genes15091170 (PMC11430887; doi:10.3390/genes15091170)
Supplement: Supplementary file 1 [file genes-15-01170-s001.zip › Figure S2 - demo_prediction.pdf]

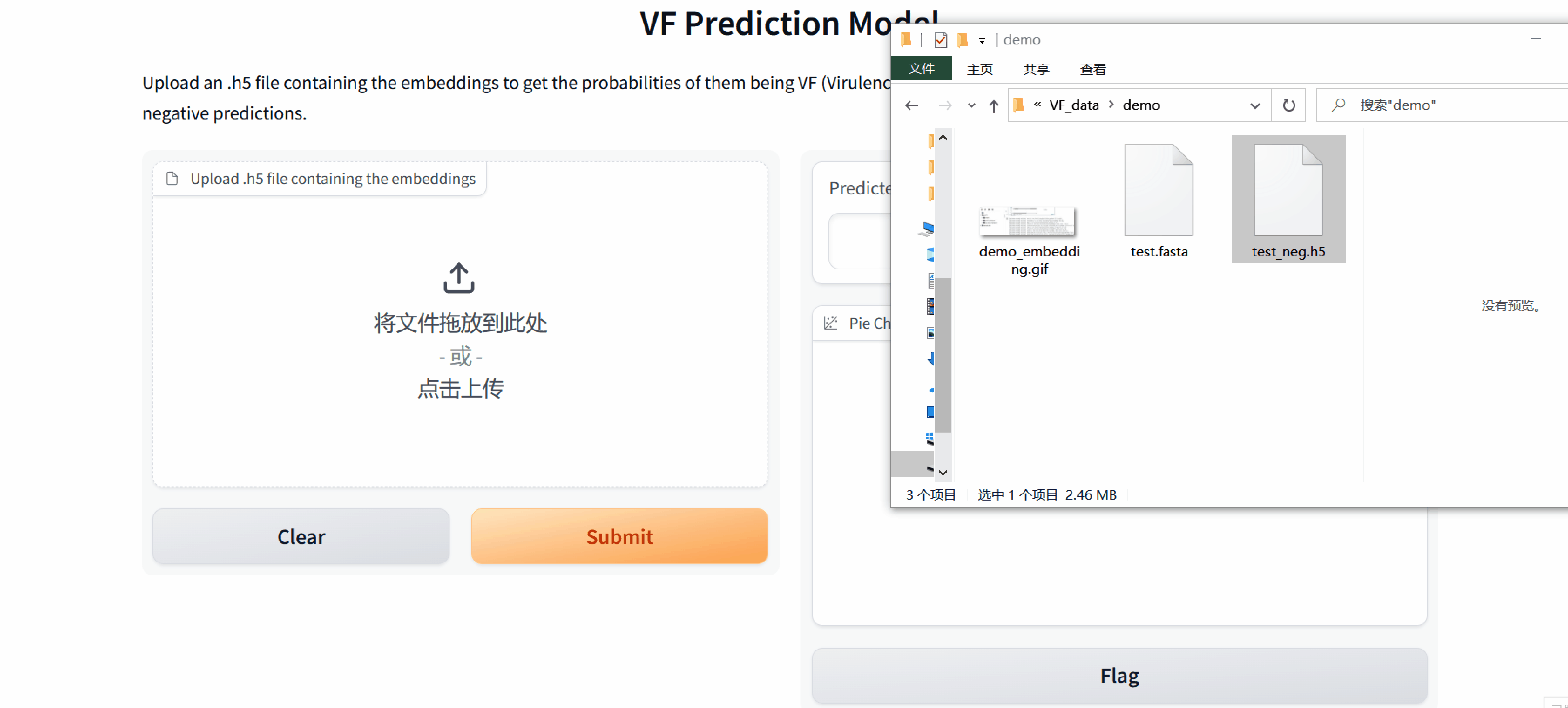
VF Prediction Mo
Upload an .h5 file containing the embeddings to get the probabilities of them being VF (Virulenc
negative predictions.
Upload .h5 file containing the embeddings
将文件拖放到此处
- 或 -
点击上传
Clear
Submit
Predicte
Pie Ch
Flag
demo
文件
主页
共享
查看
« VF_data › demo
搜索"demo"
demo_embedding.gif
test.fasta
test_neg.h5
没有预览。
3 个项目
选中 1 个项目 2.46 MB
