# Supplementary figures and images for: DTVF: A User-Friendly Tool for Virulence Factor Prediction Based on ProtT5 and Deep Transfer Learning Models
Source: Genes (Basel). 2024 Sep 5;15(9):1170. doi: 10.3390/genes15091170 (PMC11430887; doi:10.3390/genes15091170)

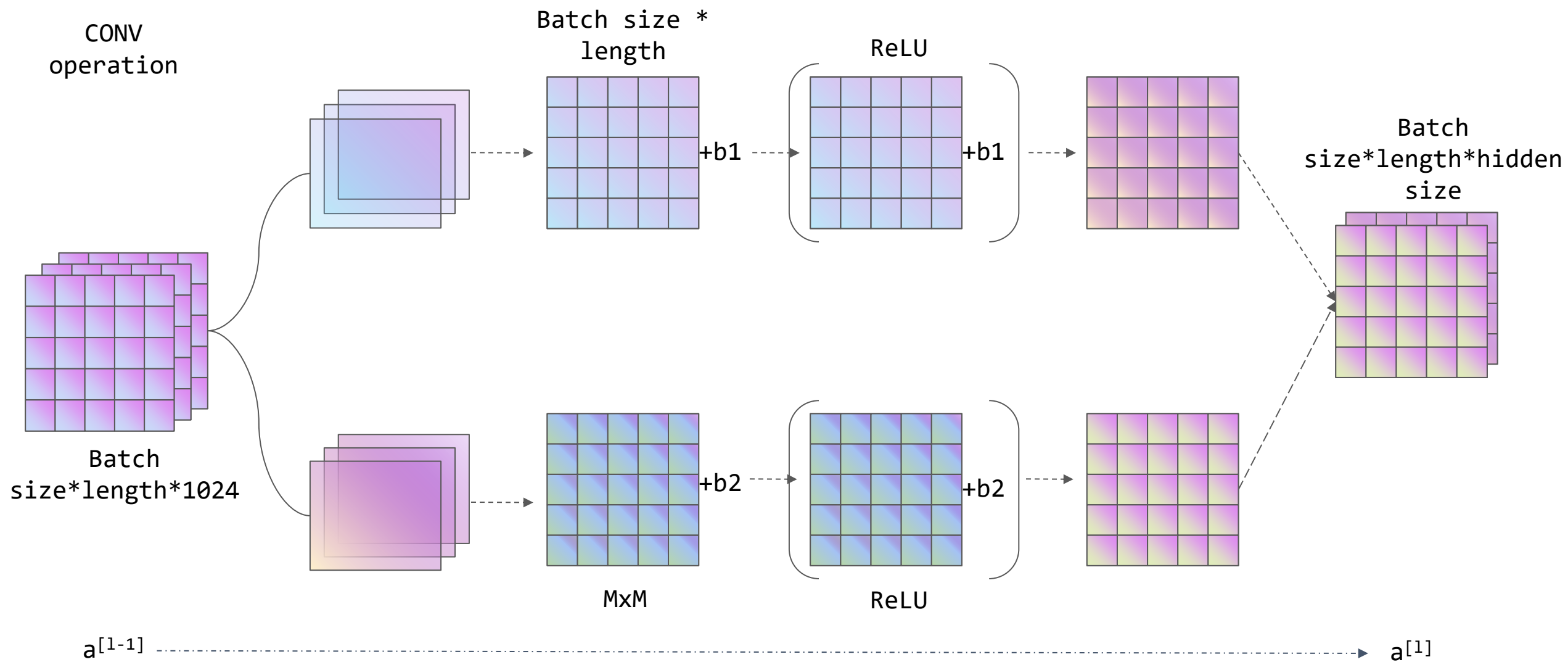

Supplement: Supplementary file 1 [file genes-15-01170-s001.zip › Figure S3.pdf]

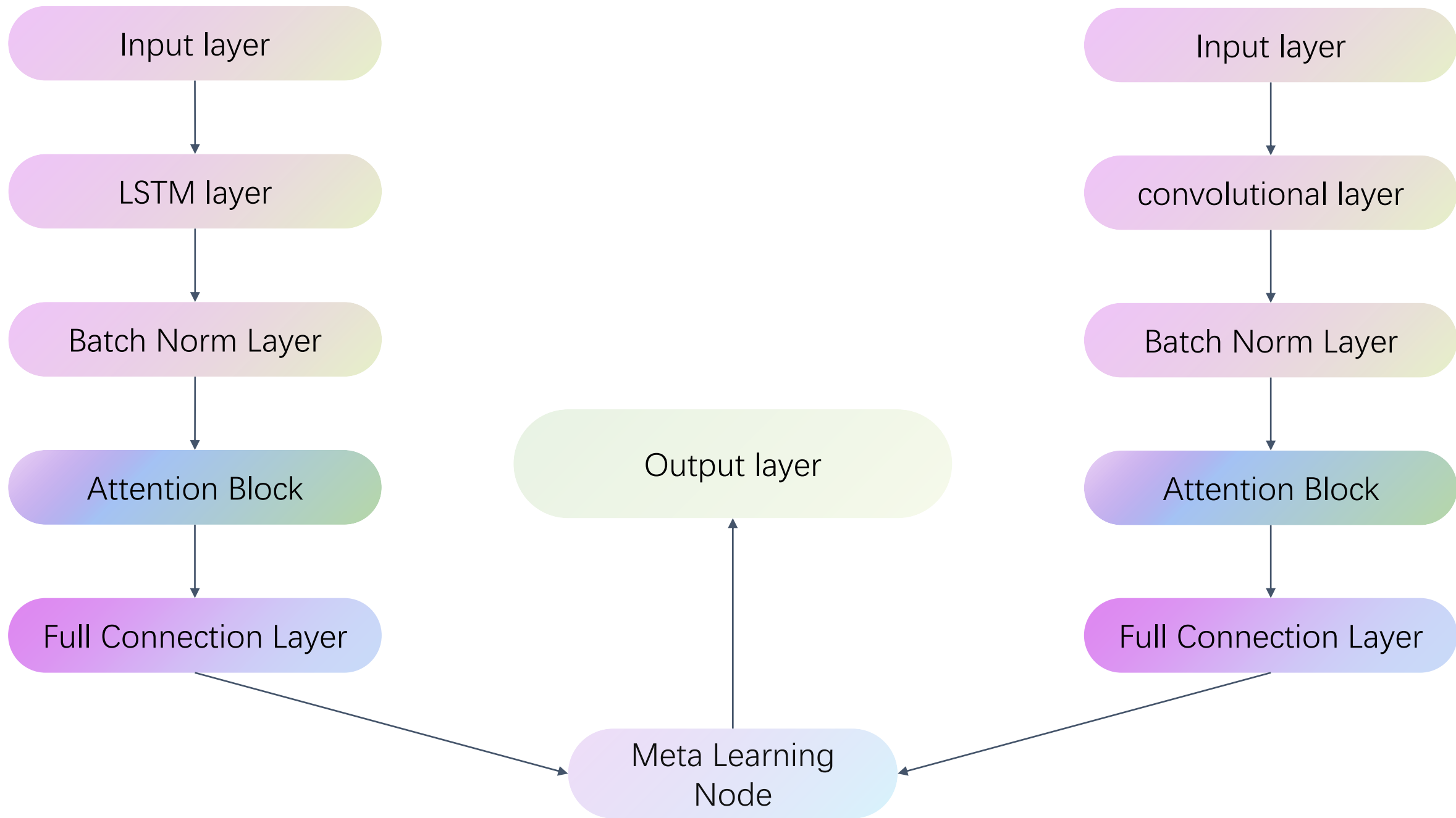

Supplement: Supplementary file 1 [file genes-15-01170-s001.zip › Figure S4.pdf]
